# Development and validation of the antecedents affecting coping questionnaire in patients with multiple sclerosis

**DOI:** 10.1186/s12883-025-04270-w

**Published:** 2025-07-01

**Authors:** Ali Dehghani, Abdolkhalegh Keshavarzi, Yasaman Pourfarid

**Affiliations:** 1 Department of Community Health Nursing, School of Nursing, Jahrom University of Medical Sciences, Motahari St, Jahrom, 7414846199 Iran; 2 Department of Nursing, School of Nursing and Midwifery, Shiraz University of Medical Sciences, Shiraz, Iran; 3 Department of General Surgery, School of Medicine, Shiraz University of Medical Sciences, Shiraz, Iran; 4 Department of Nursing, School of Nursing, Jahrom University of Medical Sciences, Jahrom, Iran

**Keywords:** Multiple sclerosis, Antecedents, Coping, Questionnaire, Validation

## Abstract

**Background:**

Development of a self-report measure of antecedents affecting coping with multiple sclerosis lead to the identification of influential factors in the coping process and the provision of health cares with a focus on providing or modifying these factors. The current study aimed to develop and validate the antecedents affecting coping scale for multiple sclerosis patients.

**Methods:**

This methodological study was conducted in two stages. In the first stage, the concept of coping antecedents was explained using qualitative content analysis approach through interview with 11 MS patients, then the initial items were extracted and the questionnaire was developed. In the second stage, reliability and validation of the questionnaire were evaluated including face, content and construct validity. The76 items in primary items pool reduced to 24 items after evaluating validity and reliability.

**Results:**

Factor analyses revealed 5 factors: social support, awareness about disease, personal opinion and attitudes, spiritual - religious beliefs, and economic - environmental factors. Internal consistency and stability of the developed questionnaire as well as respectively confirmed with 0.92 and 0.95.

**Conclusion:**

The 24 items developed questionnaires is valid and reliable for measurement of antecedents affecting coping among multiple sclerosis patients in Iran.

## Introduction

Multiple Sclerosis (MS) is a neurologic, progressive, chronic, and common disease among young adults, and most patients are 20 to 50 years old [1]. Relapsing and remitting multiple sclerosis (RRMS) is one of the most common forms of MS, affecting around 80% of patients [2]. This disease can impact both physical (e.g. fatigue, numbness, pain, blurred vision) and psychological (e.g. stress, depression) through demyelination of brain nerve fibers [3]. MS is one of the most important life-changing diseases, because it damages the best time of the individuals’ life and lead them gradually to inability [4]. This disease affects approximately 2.5 million people worldwide [5]. The prevalence of MS in Iran was estimated 29.3/ 100,000. The incidence of MS in Iran was estimated 3.4/ 100,000 (95%CI: 1.8–6.2) based on random effects model [6, 7]. The number of people with MS is about 78,890 people in Iran [6]. The results of a study indicated a relatively high prevalence of the disease in Fars province and Jahrom city compared to other parts of Iran, the reason for which may be related to climatic and geographical differences, as well as racial and cultural differences [7].

During the last decade, various studies have been conducted on the impact of MS on coping. Studies have shown that people with MS often reported problems regarding self-care, activity daily living, and coping with disease and they also became mostly unemployed or retired in consequence of this disease [8–10]. Since people with MS live with uncertainty as to the course of the disease, they need to cope with unpredictable deteriorating health, changing social and intimate relationships, and increasing support needs [10, 11]. Hence, investigating the antecedents affecting coping with disease can be effective in shifting from a medical and pharmaceutical focus to using personal experiences to promote coping and quality of life [12, 13].

Antecedents of coping with disease are the underlying factors that help patients with MS to cope with the disease, achieve better quality of life, and also positive outcomes [8]. On the other hand, patients with MS, compared with other diseases, experience a higher prevalence of affective disorders faced with various physical and psychological coping needs [14],Therefore, evaluating and measuring antecedents of coping using a standard questionnaire based on socio-cultural, economic conditions of Iranian patients can be effective in improve and reducing the need for medication.

Available questionnaires for assessment of coping styles including the coping with MS scale (CMSS) and ways of coping checklist (WCC), none of which refer to the antecedents affecting on coping with disease [15, 16]. Recently, Dehghani et al. (2017) developed MS Coping Questionnaire (MSCQ) that assesses levels of coping with MS [5]. Since the details are not available about antecedents affecting on antecedents coping in these patients. Research on antecedents affecting in coping indicated that the data were preliminary and inconclusive and more research is required in this area [14].Measuring antecedents of coping in MS patients periodically and regularly can be effective in planning health cares and helping patients to improve coping with disease and quality of life. In addition, Iranian MS patients are faced with problems lack of support for rehabilitation, medical expenses, misunderstanding of the society people about the MS etc [5, 17]. Therefore, the questionnaire used in Iran should be different from other countries to increase its validity. Hence, it is necessary to measure antecedents of coping in order to plan for improvement of quality of life and more coping with the disease [18].Currently, there is no standardized questionnaire for assessment coping antecedents that is appropriate to the socio-cultural context of Iran, and most of used questionnaires in Iran are originally developed in other countries and have not been cross-culturally adapted with Persian culture. This study aimed to develop a valid and reliable questionnaire that assesses antecedents affecting on coping with MS, MS Coping Antecedents Questionnaire (MSCAQ) in Iran.

## Methods

### Study design and participants

This methodological study was conducted in two sections using qualitative and quantitative methods from February 2023 to March 2024. The data were collected at the MS Society of Jahrom, Iran. Number of participants in the qualitative Sect. 11 MS patients and in the quantitative section were 380 MS patients. A methodological study essentially includes the following steps:

1. Defining the concepts
2. Formulating the items of questionnaire
3. developing the questionnaire
4. Testing validity and reliability of the questionnaire [19]

The phases of the study were described in the following sections.

### The first section: item generation

At this stage, two methods were used for item generation. In the first step, the concept of coping antecedents in patients MS was defined using the conventional content analysis approach. In this step, 11 patient with MS participated using purposive sampling that with this number, data saturation was achieved. Five of them were male and six were female. Their age ranged from 24 to 40 years and their disease experience varied from 1 to 16 years. The data were collected through semi-structured, face-to-face and in-depth interviews. Each interview lasted between 45 and 70 min. The interview environment of the MS society Office was selected based on the willingness of participants. Inclusion criteria for the patients were [1] diagnosis of MS disease [2], willingness to participate in the study [3], ability to express experiences and [4] absence of mental illness. Data collection was continued until data saturation. Interviews began with the general questions like “explain concerning the nature of disease and its problems” and “what factors help you to better cope with your disease?” Probing questions such as “explain more” or “give an example”, etc. were also used. All interviews were audio-recorded with the permission of the patients and transcribed verbatim in MAXQDA software, Ver10. Data were analyzed using conventional qualitative content approach based on Graneheim and Lundman model [20].

To provide rigorous and reliable data, the researchers spent quite a long time in the field, collecting the data and analyzing the data collected. Recommendations of the associates and other specialized partners in this area were used to determine the categories. Coded interviews were returned to the participants to reach agreement among the researchers and participants in the research.

In the next second, using reviewing the studies and scientific texts related to the research topic a number of items were added and items pool of were completed.

### The second section: item reduction and questionnaire validation

At this stage, in order to initial designing of questionnaire, the phrases and items extracted from qualitative content analysis and related questionnaires were assessed by the research team. Similar or duplicate items were deleted or merged. After making changes, the initial questionnaire with 73 items was prepared for validation and psychometric properties. Validation of the MSCAQ was evaluated consist of face, content and construct validity as well as reliability.

A) ** Face validity**: To assess the qualitative face validity of the questionnaire items from the viewpoint difficulty, relevancy and ambiguous were evaluated. At this stage, comments of 10 patients were applied. Quantitative face validity was calculated using item impact coefficient. The importance of each item was assessed using a 5-part Likert scale from totally importance (5 points) to no importance (1 point) by 10 MS patients. Items with an impact coefficient less than 1.5 were deleted [21].
B) ** Content validity**: to assess the qualitative content validity from a panel of 12 experts in order to evaluation of grammar, wording, item allocation and scaling of the items were used. In quantitative content validity section, content validity ratio (CVR) and content validity index (CVI) were calculated for each item. The CVR of each item was evaluated using a three-point scale including “essential”, “useful but not essential” and “not essential” by 12 experts, according to Lawshe (1975) [22] and modified table by Ayre and John Scally (2014) [23]. Thus, according to the number of 12 experts, items with a CVR value of 0.60 or higher were preserved. The CVI of each item was calculated using four-point likert scale (not relevant: 1; a little relevant: 2; somewhat relevant: 3; and extremely relevant: 4). The Waltz and Bussels criteria was used for calculate of CVI value [24]. Thus, Items with a CVI of 0.79 or higher were preserved.
C) ** Initial reliability**: Before calculating the construct validity, at this stage, the internal consistency and inter-item correlation of the questionnaire in order to determine correlation between items and as well as items with whole questionnaire was assessed with 50 MS patients.
D) ** Construct validity**: to determine the construct validity of the questionnaire was used exploratory factor analysis (EFA) method. Factor analysis assesse the inter relationship between items and categorizes the inter-related items [25]. In the factor analysis, Principal Component Analysis (PCA) for extraction of factors, Kaiser- Meyer- Olkin sampling index (KMO) for sampling adequacy, Bartlett’s Test for determination appropriateness of the factor analysis model, factor rotation (varimax rotation) for simplify and interpretability of the factor structure and as well as the scree plot and eigenvalues for the number of factors was used. The number of samples needed for exploratory factor analysis designated between 3 and 10 individuals per items [26]. In some studies, the minimum sample size for exploratory factor analysis is determined 300 individuals [27]. In this study, for each item the 10 samples were considered (380 MS patients) (Table 1). The study subjects were recruited using convenience sampling. Inclusion criteria were having a definite diagnosis of MS and desire to participate in the study. The minimum factor loading of 0.3 was considered for maintaining item in factor extraction.
E) ** Final reliability**: At this stage, the internal consistency and stability of the questionnaire was assessed using Cronbach’s alpha coefficient anding test-retest technique. A Cronbach’s alpha Coefficient of 0.7 or higher was considered optimal [28]. Then, correlation between scores of test and retest was calculated by intraclass Correlation Coefficient (ICC). In order to check the stability of the questionnaire, 30 MS patients completed the questionnaire twice with time interval a two-week [29].The ICC of 0.8 or higher was considered acceptable stability [30].

### Statistical analysis

Statistical analyses were conducted using the SPSS version 21.0. Descriptive analysis test, factor analysis, Cronbach’s alpha, and ICC were used for data analysis in the study quantitative section.

## Results

### The first section

In this section, the concept of coping antecedents in MS patients was explained based on the literature review and patients’ experiences using the conventional content analysis. Of the 11 MS patients in the qualitative part, 5 men and 6 were women. The mean age of the participants in the qualitative part was 33.2 years, and the average dealing duration was 10.5 years. Based on the results of the qualitative part of the study, coping antecedents in MS was found a relatively subjective and multidimensional concept which affected by factors including social support, awareness about disease, personal opinion and attitudes, religious - beliefs factors, and economic - environmental factors (categories extracted from the qualitative part of the study).

In this section, the findings of the conventional content analysis and literature review were used to generate an item pool for the MSCAQ. In this part of the study, related questionnaires were also reviewed and items related to the current questionnaire were extracted. Thus, 76 items were formed in the initial pool. The initial pool of items was reviewed for overlapping, removing, reviewing, or merging items in two sessions. Thus, 11 items deleted due to overlapping. Finally, the initial questionnaire with 73 items in five point Likert very low [1], low [2], average [3], high [4] and very high [5] were entered into validation section.

### The second section

**Fig. 1:**
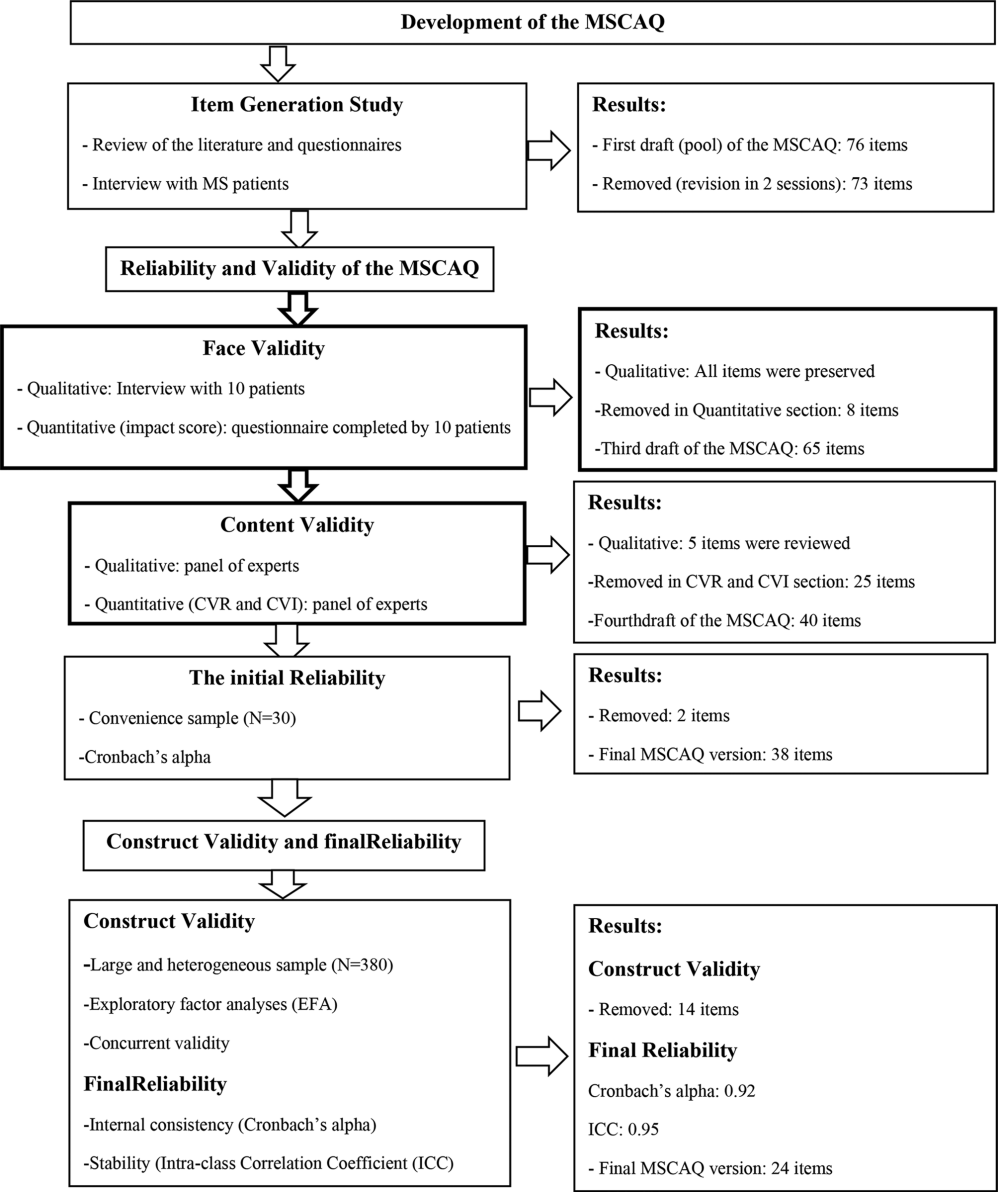
Flow diagram of the development and validation of the coping antecedents Questionnaire in MS patients (MSCAQ)

A) ** Face validity**: In qualitative examination of face validity, no item was deleted. In quantitative face, validity 5 items were revised due to impact coefficient of less than 1.5.
B) ** Content validity**: In the qualitative content validity, 5 items were reviewed based on comments of expert’s panel. In the quantitative content validity, 20 items were removed due to CVR less than 0.60. In addition, the number of 5 items were deleted because CVI of less than 0.79.
C) ** The initial reliability**: The internal consistency of the whole MSCAQ with Cronbach’s alpha coefficient was calculated 0.88 in 50 samples. In addition, 2 items “going on a travel makes me cope with disease” and” having my problems heard by others is effective in coping to my illness” were removed due to correlation of less than 0.3 with whole MSCAQ. Thus, 38 items remained for the MSCAQ.
D) ** Construct validity**: 38 items of the MSCAQ were analyzed to a principle-components factor analysis trough varimax rotation. KMO index for sampling adequacy was adequate 0.917. The Bartlett’s test shows significant relationship between the items with p-value = 0.001. The results factor analysis with varimax rotation shows five factors with eigenvalues greater than 1.0. The scree plot also extracted five factors for the MSCAQ scale. Table 2 shows the eigenvalues, percentage of variance for each factor and factor loadings for the items that met retention criteria.
  Following perform of construct validity the number of 14 items were removed because factor loading less than 0.3.After the construct validity and factor analysis, 24 items with five factors including factor one “social support” (8 items), factor two “awareness about disease” (3 items), factor three “personal opinion and attitudes” (6 items), factor four “spiritual - religious beliefs” (3 items), and factor five “economic - environmental agents” (4 items) remained for the MSCAQ.The five rotated factors explained totally 57% of the total variance (Fig. 1).
E) **The final reliability**: The Cronbach’s alpha coefficient for 24 – items MSCAQ was 0.92 that indicates acceptable internal consistency. The ICC between test and retest measurements for 24 – items MSCAQ was 0.96 that indicates desirable stability of MSCAQ during the time. As well as Cronbach’s alpha and ICC was determined for five factors (Table 3).

**Table 1:** Demographic characteristics of the samples in EFA section (*N* = 380)

Variables		Mean ± Standard deviation
Age (years)	Mean (SD)	34.17 ± 10.12
Duration of MS (years)	Mean (SD)	10.32 ± 5.54
		**N (%)**
Gender	male	160 (42.1)
	female	220 (57.9)
Educational level	Under diploma	93 (24.5)
	diploma	165 (43.5)
	Upper diploma	122 (32)
Number of recurring during the past year	No recurring	235 (61.9)
	once	81 (21.3)
	More than 2 times	64 (16.8)

**Table 2:** Results of a principal components analysis of the 24 coping antecedents items (*N* = 380)

Subscales	Item	Factors				
		1	2	3	4	5
Social support	Talking to family members makes me cope with the disease.	**0.701**	0.201	0.234	0.167	0.123
	Understanding of community people is effective in my coping with the disease.	**0.705**	0.102	0.701	0.213	0.123
	Family financial support makes me cope with the disease.	**0.687**	0.298	0.157	0.233	0.145
	Sharing positive experiences with MS patients is effective in my coping with the disease.	**0.712**	0.231	0.222	0.154	0.255
	To coping with the disease, I need to use multidisciplinary teams including nurse, psychologist, caregiver and psychiatrist.	**0.632**	0.105	0.198	0.166	0.244
	Proper communication by the treatment team is effective in my coping with the disease.	**0.680**	0.237	0.245	0.266	0.231
	Having access to mobility aids like wheelchairs, public transportation for MS patients is effective in my coping with the disease.	**0.556**	0.132	0.165	0.234	0.143
	Easy access to the treatment team is effective in my coping with the disease.	**0.587**	0.123	0.287	0.234	0.145
Awareness about disease	The accuracy of the treatment team in diagnose the disease is helpful in me coping with the disease.	0.211	**0.745**	0.231	0.234	0.243
	Increasing my awareness and knowledge about disease makes me coping with the disease.	0.234	**0.621**	0.299	0.286	0.211
	Awareness of community people and family members about MS is effective in my coping with the disease.	0.132	**0.633**	0.233	0.287	0.111
Personal opinion and attitudes	I believe that feeling guilty and blaming oneself are influential in coping with the disease.	0.122	0.234	**0.614**	0.235	0.233
	When I compare MS to other chronic illnesses like cancer, I had better can coping with the disease.	0.144	0.244	**0.555**	0.123	0.213
	I believe that being happy and having high spirits will help in my coping with the disease.	0.188	0.198	**0.524**	0.167	0.211
	I believe that distraction and entertainment with favorite things in my coping with disease are effective.	0.244	0.111	**0.501**	0.245	0.198
	I believe that independence in daily activities is effective in my coping with the disease.	0.288	0.122	**0.498**	0.235	0.276
	I believe that having a daytime activity is effective in coping to my illness.	0.133	0.234	**0.503**	0.133	0.213
Spiritual - religious beliefs	Belief in God is effective in coping with my illness.	0.122	0.260	0.239	**0.601**	0.263
	Accepting the disease as a divine test makes me compatible with the disease.	0.188	0.109	0.233	**0.544**	0.223
	Participation in religious ceremonies and prayers makes me compatible with the disease.	0.201	0.204	0.238	**0.530**	0.133
Economic - Environmental agents	The multiple attacks in this disease are influential in coping with the disease.	0.213	0.125	0.277	0.210	**0.611**
	Living facilities (such as elevators, toilets, etc.) are effective in my coping with the disease.	0.267	0.138	0.101	0.125	**0.654**
	A quiet and stress-free environment makes my coping with disease.	0.234	0.238	0.202	0.108	**0.543**
	Employment and eliminate occupational concerns are effective in my coping with the disease.	0.263	0.133	0.256	0.189	**0.608**
Eigenvalue		**10.230**	**7.320**	**4.211**	**1.432**	**1.123**
% of variance		**23.600**	**12.300**	**9.250**	**8.455**	**3.395**

**Table 3:** The cronbach’s alpha and ICC values for MSCAQ scale and its factors

Factors	Subscales	Number of items	Internal consistency	Stability
1	Social support	8	α = 0.91	ICC = 0.89
2	Awareness about disease	3	α = 0.79	ICC = 0.92
3	Personal opinion and attitudes	6	α = 0.83	ICC = 0.88
4	Spiritual - religious beliefs	3	α = 0.76	ICC = 0.94
5	Economic - environmental agents	4	α = 0.81	ICC = 0.87
	MSCAQ	24	α = 0.92	ICC = 0.95

## Discussion

The present study reported the stages of designing and developing a scale for evaluating antecedents affecting on coping in MS patients and the findings indicated satisfactory psychometric properties for the questionnaire. The final version of the MSCAQ has 24 items in five domains contains: social support, awareness about the disease, personal opinion and attitudes, spiritual - religious beliefs, and economic - environmental agents.

The first domain of the MSCAQ scale, social support, consists of eight items. The items of this domain showed that having social support is very effective in coping patients with disease. Social networks contains family, spouse, friends, peer patients, employees, and relevant professional organizations are as a source of support so that their support can reduce negative outcomes caused by MS and help the patients to better cope with their disease. Social support for coping with disease is a crucial issue in adults with MS disease. It can contribute to drug adherence and the outcomes [31]. The results of the study by Krokavakova et al., showed that better emotional, psychological and social support from family and friends is directly related to improving mental health and patients coping to MS [32]. On the other hand, patients who have less social support are more face to mental health problems, especially stress, and the severity of the disease is higher; this can have effects on the patients’ coping with disease [14]. In the patient’s assessment with MS, patients who did not receive adequate social support did not have acceptable coping with disease. In addition, the coping behavior of patients with MS was connected to social support, especially support by family, friends, or other patients with MS [31].

The second domain of the MSCAQ was awareness about disease that had three items. Learning and knowledge about the disease and its treatment modalities gradually reduces the patients’ fear and enables them to cope with the conditions. The patients in present study tried to cope with their disease using gathering information and increasing awareness about the MS and its treatment and outcomes. The quality of information provided by health personnel, particularly at the time of disease diagnosis, is related to the ability to better coping in patients [33]. In a study by Wilski et al., the results showed that community people awareness about MS disease are effective in coping behaviors and self-management of patients [34].

The third domain of the MSCAQ was personal opinion and attitudes with six items. The personal opinion and attitudes like high spirits, positive thinking etc. are effective important factors for coping with disease. In addition, the psychological studies show that personal opinion and attitudes are an important factor in determining health, having successful functioning and social interactions [35]. According to research of Finstad et al., positive thinking reduces negative emotions and increases positive emotions and coping behavior [36].

The fourth domain of the MSCAQ was spiritual - religious beliefs that had three items. The participants believed the MS disease as a god’s test. In several studies, the positive role of religion and hope on eternal power of god in coping has been mentioned [37, 38]. The results of the Burlacu et al., showed that religiosity play an important role in both quality of life and health status of hemodialysis patients [39]. The results of a study in Iran on chronic diseases showed that religious beliefs not only are effective in coping with the disease, but play an important role in patients’ lifestyle, and create a sense of purposefulness, increase the patients’ motivation to the treatment regimen and are used as a sedative agent by patients [40].Also Hassani et al., found that spiritual - religious beliefs can serve as a coping mechanism for end stage renal failure patients to enhance their health related quality of life [41]. According to MS patients’ experience, the deep mental concerns about disease and also the severe physical problems need to strengthen the spiritual - religious dimensions. Therefore, the nurses and other health professionals need to effectively and successfully integrate the spiritual care with their professional performance for improve coping with disease and quality of life.

The fifth domain of the MSCAQ was economic - environmental agents that had four items. Because the progressive nature of some types of the MS disease, many patients lose their employment that leading to low productivity and incomes of life and have negative effects on the patient’s coping [42].Also, providing conditions such as appropriate living facilities and a quiet and stress-free environment can positively effect on patients’ coping with disease. In particular, patients who left work due to their MS were found had a longer disease duration and progressive course, reported greater disability and fatigue, and worse performance in coping with disease [43]. The meta-analysis findings of Zhao et al., showed that living environment and environmental conditions have been implicated in many gastrointestinal and neurological diseases such as MS, Parkinson’s, etc., and have adverse effects on physical and mental health and coping [44].In a meta-analysis performed by Tang et al., A direct linear relationship was found between stressful environmental conditions and worsening of multiple sclerosis [45].

The present study has several strengths like the MSCAQ scale was developed the both inductive and deductive approach and using psychometrics properties including face, content, and construct validity, and as well as conducting face-to-face interviews to collect more complete and valid data. In addition, the MSCAQ is a short – form (24 items) questionnaire that can be responded by MS patients in less than 15 min. The biggest strength of present study was the development of a context-bound antecedents affecting on coping to assess Iranian MS patients coping antecedents. However, there are a number of limitations such as suffering questionnaire of self-report scales to collect data and the lack of measures derived from already validated questionnaires to investigate the concurrent/divergent validity of the new developed questionnaire.

## Conclusion

MS Coping Antecedents Questionnaire (MSCAQ) is a valid and reliable questionnaire for evaluating the coping antecedents in patients with MS and can be used in future studies.

## Data Availability

Data is not and will not be made available elsewhere. Further data set could be obtained on request if required through corresponding author with email: ali.dehghani2000@ gmail.com.
